# Meralgia Paresthetica—An Approach Specific Neurological Complication in Patients Undergoing DAA Total Hip Replacement: Anatomical and Clinical Considerations

**DOI:** 10.3390/life14010151

**Published:** 2024-01-20

**Authors:** Jozef Almasi, Richard Ambrus, Boris Steno

**Affiliations:** 1 Department of Orthopaedics, Nemocnica Bory Penta Hospitals International, I. Kadlecika 2, Lamac, 841 03 Bratislava, Slovakia; jozef.almasi@pentahospitals.com; 2 II. University Department of Orthopaedic and Trauma Surgery, University Hospital Bratislava, Faculty of Medicine, Comenius University Bratislava, Antolska 11, Petrzalka, 851 01 Bratislava, Slovakia; boris.steno@fmed.uniba.sk

**Keywords:** direct anterior approach, meralgia paresthetica, lateral femoral cutaneous nerve injury

## Abstract

*Introduction:* Mini-invasive surgical (MIS) approaches to total hip replacement (THR) are becoming more popular and increasingly adapted into practice. THR via the direct anterior approach (MIS DAA) has become a rather controversial topic in hip arthroplasty literature in the last decades. Our retrospective observational study focuses on the prevalence of one approach-specific complication—lateral femoral cutaneous nerve (LFCN) iatrogenic lesion—and tries to clarify the possible pathogenesis of this injury. *Methods:* This is a retrospective single-cohort observational single-center and single-surgeon study. Our patient records were searched for the period from 2015 to 2017—after a safe period of time after the learning curve for MIS DAA. All intra- and post-operative lesions of the LFCN were recorded. Lesion of the LFCN was confirmed by a neurological examination. Minimum patient follow-up was 2 years. *Results:* This study involved 417 patients undergoing single-side THR via MIS DAA. Patients were examined on follow-up visits at 6 weeks, 6 months, 1 year, and 2 years after surgery. There were 17 cases of LCFN injury at the 6 weeks early follow-up visit (4.1%). All cases of clinically presenting LFCN injury resolved at the 2-year follow-up ad integrum. *Discussion:* Possible explanations of such neurological complications are direct iatrogenic injury, vigorous traction, hyperextension, or extreme external rotation of the operated limb. Use of a traction table or concomitant spinal pathology and deformity also play a role. Prevention involves stepwise adaptation of the approach during the learning curve period by attending cadaver lab courses, rational use of traction and hyperextension, and careful surgical technique in the superficial and deep fascial layers. Dynamometers could be used to visualise the limits of manipulation of the operated limb. *Conclusions:* Neurological complications are not as rare but questionably significant in patients undergoing THR via the DAA. Incidental finding of LFCN injury has no effect on the functional outcome of the artificial joint. It can lead to lower subjective satisfaction of patients with the operation, which can be avoided with careful education and management of expectations of the patients.

## 1. Introduction

Anterior approach to the hip joint was first described by Hueter in the late 19th century [1]. The modern direct anterior approach is a modification of that technique, utilising the distal part of the Smith-Petersen interval between the sartorius and tensor fasciae latae muscles superficially and between the rectus femoris and gluteal muscles in the deeper layers, without cutting into or otherwise damaging them [2]. It is thus the only truly intermuscular and interneural approach, avoiding dissection and injury to the muscle envelope of the hip joint, especially the gluteal girdle. Among the advantages of using this approach for total hip replacement when compared to the lateral and posterior approaches are faster recovery time and rehabilitation, lower blood loss, lower pain scores, and lower rate of hip dislocation, as well as shorter length of hospital stays [3]. Shifting the incision and interval further anteriorly and proceeding in a muscle sparing minimally invasive fashion spares the muscle envelope of the hip joint, reducing posterior instability and allowing return to full function earlier [4].

On the other hand, the disadvantage and biggest hurdle to the widespread adaptation of the direct anterior approach is a longer learning curve and higher complication rate in this period [5,6]. The learning curve is completed by about 100 cases [7]. The adverse events that tend to occur during this period at a higher rate include implant malposition, periprosthetic fracture, and longer operative time [5]. Neurological complications may occur in any of the studied approaches; however, in the direct anterior approach, the most common neurological complication is injury to the lateral femoral cutaneous nerve (LFCN) or its branches [8,9]. This is a neurological complication specific to the anterior approach, related to the location of the skin incision and preparation of the superficial layers of this surgical approach. Literature regarding the rate of this complication vary, with some authors reporting 81% [10,11,12,13]. The other nerves at risk are the femoral nerve with reported incidence of 0.34% in the literature, and branches of the superior gluteal nerve distributing to tensor fasciae latae muscle (TFL) resulting in fatty atrophy of tensor fasciae latae muscle, with insufficient hard data on its incidence—most studies are cadaveric analyses and show a theoretical risk of injury [8,9,14,15].

**Figure 1:**
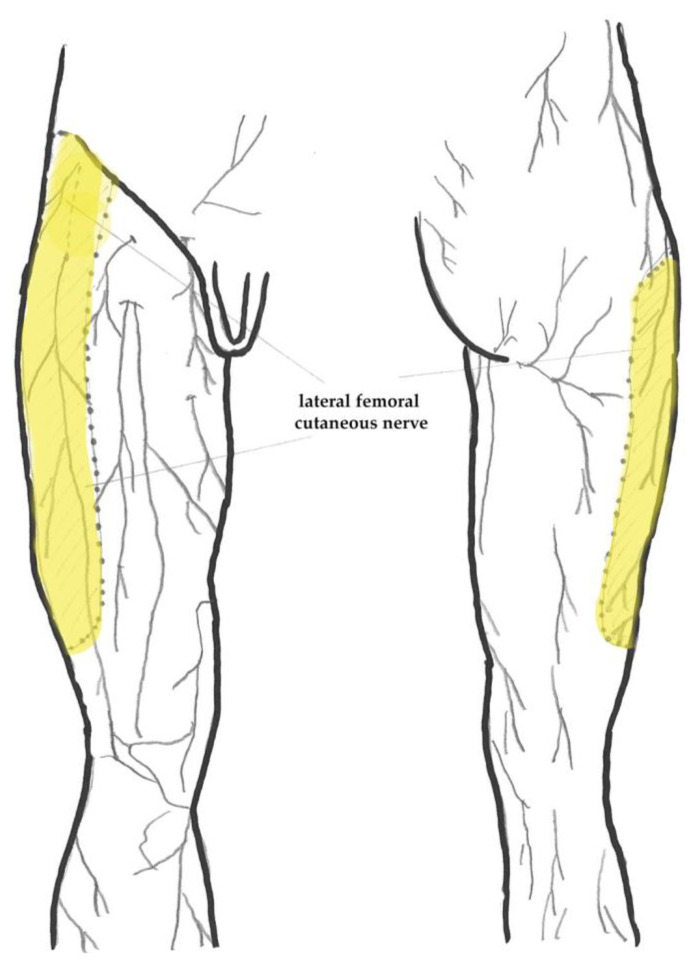
Anatomical diagram depicting distribution of skin innervation of the respective cutaneous nerves. Highlighted (yellow) is the area of distribution of the LFCN dermatome from the front (**left**) and back (**right**). Image redrawn from Gray’s Anatomy of the Human Body, 1918, licensed under CC0 1.0 [25].

**Figure 2:**
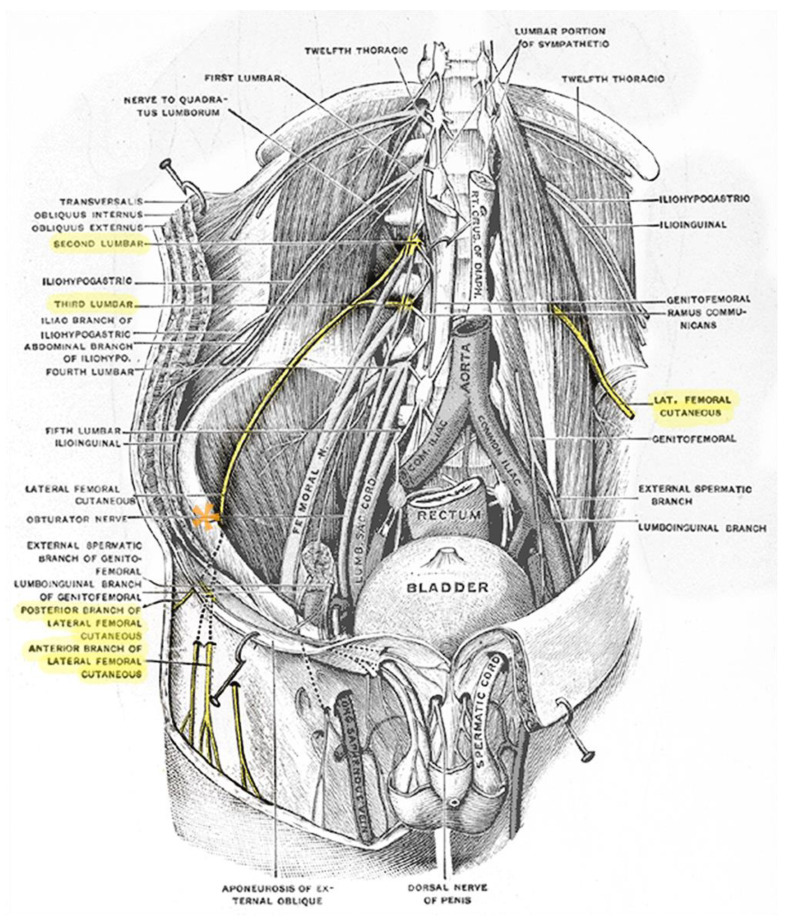
Anatomical illustration of the abdominal and intrapelvic course of the highlighted lateral femoral cutaneous nerve (LFCN (yellow)) exiting from the second and third lumbar roots, emerging through the psoas muscle and around the anterior superior iliac spine (asterisk), underneath the inguinal ligament into the superficial suprafascial layers of the anterolateral thigh. Plate 824 adapted from Gray’s Anatomy of the Human Body, 1918, licensed under CC0 1.0 [25].

The lateral femoral cutaneous nerve distributes sensation to an area of skin roughly the size of the patients’ palm on the proximal lateral aspect of the thigh, as shown in Figure 1. Its root origin is from the second and third lumbar spinal nerve, coursing into and across the psoas major muscle emerging laterally, then inferiorly and laterally along the iliacus muscle from where it generally runs toward the medial edge of the anterior superior iliac spine (ASIS), as shown in Figure 2 [16,17]. The exact course of the lateral femoral cutaneous nerve in the distal intrapelvic course as it nears the anterior superior iliac spine and the inguinal ligament is hard to pinpoint [18,19,20,21,22]. The literature provides information on variation mainly at two levels—the location of the nerve at the level exiting the pelvis and the variation of the level and type of branching of the lateral femoral cutaneous nerve into its final divisions [23]. It may also be absent on one side and/or its dermatome may be supplied by the ilioinguinal nerve or branches of the anterior femoral cutaneous nerves instead [24].

The variation at the level before exiting the pelvis relates mainly to the relationship with the anterior superior iliac spine. This is relevant to the risk of iatrogenic injury during surgical procedures or as a predictive factor for mechanical cause of meralgia paraesthetica. In a meta-analysis of 1473 specimens, seven types of various locations where the lateral femoral cutaneous nerve crosses the pelvic brim were found—medial to, over, lateral to, or through the anterior superior iliac spine, through or over the inguinal ligament, and through the sartorius muscle. Most common variation showed the nerve exiting under the inguinal ligament and medial to the anterior superior iliac spine and sartorius muscle [23].

Mononeuropathy of the lateral cutaneous nerve of the thigh was first described by Hager in 1885 as “traumatic neuritis” of the lateral femoral cutaneous nerve at the point of exit from the pelvis. In that clinical case, the patient presented with numbness and ‘neuritic’ pain of the lateral thigh after sustaining an injury on a dance floor following getting their hip checked [19,26]. In this case, Hager decided to treat the patient with surgical neurectomy. This syndrome of burning, tingling, numbness, and pain in this area of skin was named meralgia paresthetica (MP) by Roth (sometimes also referred to as Bernhardt-Roth syndrome) [27]. The syndrome can be idiopathic; however, its aetiology, according to literature, is generally mechanical nerve entrapment or direct injury, such as from an external force ranging from contusion to fracture of the surrounding skeleton (e.g., acetabular, pelvic ring fractures) or iatrogenic during a surgical procedure in the anterolateral proximal thigh region, e.g., total hip replacement through the direct anterior approach [19,28]. Tight clothing has also been implicated in the existing literature as the culprit in causing or exacerbating meralgia paresthetica [29]. Iatrogenic injury is also possible during other surgical procedures, such as acetabular fracture repair, harvesting of the iliac crest bone graft, or non-orthopaedic procedures, including inguinal hernia surgery, appendectomy, indwelling catheters for the purpose of continuous anaesthesia, and aesthetic abdominoplasty [30,31,32]. According to literature, treatment is usually conservative with symptomatic therapy and patient education until spontaneous remission, adjusting garments or reducing weight [33]. With persistent symptomatology, prescriptions range from analgesics to anticonvulsants (e.g., gabapentin) [34]. Local nerve block or glucocorticoid injection can provide temporary relief [35,36]. In rare cases, surgery might be chosen —decompression of the nerve, usually the inferior attachment of the inguinal ligament to the superior anterior iliac spine or the tendinous sartorius origin [37,38]. The most definitive procedure is nerve transection at the point of exit from the pelvis, providing pain relief but also permanent anaesthesia of the dermatome [34,39].

The relevant topographic anatomy in the direct anterior approach, with regard to this complication, considers the superficial anatomy of the region of the upper thigh—surface landmarks defining the region of interest comprise: cranially, the inguinal crease (Holden’s line) and the palpable bony anterior superior iliac spine; laterally, the bony protuberance of the greater trochanter; and medially, the sartorius and rectus femoris muscles. The skin of the lower limb is generally thicker than that of the upper limb as an adaptation to weight-bearing. In the anterior region of the upper thigh, however, the skin is less thickened than posterior thigh and the buttocks, which bear weight during sitting and consequently are relatively thick. The hypodermis of the lower limb consists of thin areolar tissue with variable quantity of fat and becomes acrally thinner. Superficial veins and cutaneous nerves connect to the subcutaneous tissue with thin adventitial fibres to prevent their displacement during movement. Near the inguinal ligament, the areolar tissue forms distinct layers and is thicker in the inguinal region where two layers enclose the superficial inguinal lymphatic nodes, long saphenous vein, and other smaller vessels, blending together overlying the saphenous opening where the vessels perforate it, giving the name cribriform fascia. The superficial fascia of the thigh is a continuation of the abdominal fascia and often shows two distinct layers. The more superficial fatty layer is the continuation of Camper’s fascia, while the deeper membranous layer is an extension of the fascia of Scarpa [40]. Over the inguinal ligament they fuse to the deep fascia—fascia lata—that forms a tough circumferential ‘stocking-like’ structure over the muscles. Fibrous septa pass deep to their bony attachments, forming functional muscular compartments, serve as additional areas of attachment or function as accessory tendons [24]. It is along these fascial planes that the lateral femoral cutaneous nerve is susceptible to compression or injury, whether during the course of the main branches over the deep fascia or at the points of perforation of superficial layers by the terminal branches supplying the skin [16,17,18,19,20,21,22].

## 2. Materials and Methods

We conducted a retrospective database search of all patients undergoing total hip replacement using the direct anterior approach performed by a single surgeon (AJ). The study period was chosen as 2015–2017, well after implementation of the approach by the department, to avoid the period of learning curve, which the author considers to be more than 100 cases. Basic demographic description of the population was noted.

We searched for and noted patients with subjectively significant symptomatology of a lateral femoral cutaneous nerve lesion at the point of discharge or at any follow-up visit. Patients are followed up and queried for any discomfort at 6 weeks, 6 months, and 12 months after surgery and then once yearly. Clinical complaints of pain, burning sensation, tingling, numbness, or a feeling of discomfort were included. All patients with these symptoms at the earliest follow-up were referred to neurological clinical examination to confirm the diagnosis of meralgia paresthetica as per their standard clinical practice. Peri-incisional events, such as wound inflammation or other complication that could mask as meralgia paresthetica, were differentiated, and excluded.

Only patients with 2 years of follow-up at the end of the study period were included. Any concomitant peri- or post-operative complications or changes in the general neurological status of the patient were noted.

The surgical technique used during the study period was standardised for all patients undergoing total hip replacement. The single senior surgeon performed all procedures using the direct anterior interval of Smith-Petersen. The precise variation of the approach was the Innsbruck technique [19]. The patient lies in a natural supine position on a regular positioning table, with the pelvis located over the table break in preparation for hip hyperextension, without bolsters or bumps underneath. In patients with significant central obesity, their abdominal pannus is retracted away using adhesive tape to avoid interference with exposure. After draping, a skin incision is made in a slightly dorsally sloped, longitudinal fashion using the surgical scalpel, starting from a point 2 fingerbreadths lateral and distal from the anterior superior iliac spine, continuing toward the lateral knee and caput fibulae, making a 10–12 cm cut, depending on the size of the patient. Sharp dissection of the layer of subcutaneous fat continues using monopolar electrocautery while retracting the medial edge of the wound until signs of first fascial tissues are visible, the lateral edge of the wound falls open with gravity. Targeted haemostasis is achieved continuously during dissection. Using a periosteal elevator, gentle blunt scraping motion of the superficial fascial layer toward the edges reveals the true fascia and the epimysial covering of the TFL, which is distinguished from the more medial sartorius muscle due to its red muscle belly, as opposed to the whitish, tendinous sartorius. The lateral femoral cutaneous branches are incorporated in-between this fascial layer and the superficial, more adipose fascia. Further deep, the dissection continues with opening the tensor fascia, blunt dissection of the muscle belly and retraction laterally, ligation of the ascendent branches of the lateral circumflexa femoris vessels, and retraction of the caput reflexum of the rectus femoris muscle (capsulectomy). Retractors placed around the femoral neck and anterior rim of the acetabulum. After the neck cut and extraction of the femoral head and osteotomed neck, stepwise acetabular reaming is done with an off-set or a straight handle until desired diameter is reached. A pressfit technique is used to implant the definitive socket and liner. After releasing the posterior capsule and pubofemoral ligament from the femoral neck, a femoral lift and limb hyperextension are done. This allows a clear view of the femoral canal, and stepwise broaching is done using a single or double off-set broach handle. When stability is reached with a trial broach, the system is tested with trial heads until full system stability is satisfactory without a risk of impingement or dislocation. The final cementless implant is put in place, and the joint is reduced. In the end, after one final haemostasis control, the wound is closed in layers—fascia, subcutaneous fat, and skin. Sometimes, both the deep (true) fascia and the superficial fascia are closed shut to avoid dead space and haematoma formation. Implants are checked on a post-operative x-ray, excluding under-sizing or periprosthetic fractures. Mobilisation with full weight bearing on elbow crutches starts on post-operative day one with regular analgesia protocols. Patients are discharged to home environment when fully mobilised and followed up at intervals of two weeks, six weeks, six months, one year after surgery, and then once every two years.

## 3. Results

In our retrospective database review, we identified 417 total hip replacement procedures using the minimally invasive direct anterior approach with sufficient follow-up of 2 years. The majority of the studied population was female (284 or 68.1%). The average age at surgery was 62.1 years. Follow-up in the studied cohort was 2 years minimum, or until the symptoms persisted.

At the early follow-up visit at 6 weeks, 17 cases of clinically confirmed meralgia paraesthetica were identified, resulting in our rate of the meralgia paraesthetica complication of 4.1% in this population. All described it as a discomfort. Overall, 12 had decreased sensation over the dermatome of the lateral femoral cutaneous nerve, 5 had tingling/paraesthesia; of these, 1 described it as a waxing and waning painful burning sensation. All patients were educated about the diagnosis and watched for the clinical course of the symptomatology. No other intervention was indicated. The cases are summarised in Table 1.

Of these cases, 10 (58.8%) resolved themselves by the 12-month follow-up visit. All 17 cases of meralgia paraesthetica in this population resolved spontaneously by the 24-month follow-up visit (Table 1). No objective nor subjective functional issues of the operated hip were encountered, and patients returned fully to their activities of daily living. No residual neurological symptoms or numbness in the affected dermatome were found.

## 4. Discussion

The anterior approach to the hip joint has an extensive history in the world of orthopaedic surgery since the description by Carl Hueter. The earliest adaptations by Smith-Petersen and Judet in the beginning of the 20th century have been shown the application in paediatric orthopaedics, hip resurfacing, and femur fractures [1,2]. In the modern era of total hip arthroplasty, the approach has evolved with the Innsbruck technique for total hip replacement and, more significantly, later by Joel Matta [41,42]. The latest evolution of the approach is using a transverse skin incision in, or just below, the groin crease, called the bikini incision [43,44]. Evolution of the approach went hand in hand with the development of new minimally invasive surgical instrumentation and operative tables.

As the direct anterior approach is still evolving, this poses a hurdle in the interpretation of the literature and the reported complications. Discrepancy in the specific variation of the approach used by the reporting surgeons is a contributing factor in the wide range of complication rates in the literature. The specific number of the rate of meralgia paraesthetica ranges up to 81% [8,9,10,13,45].

Possible explanations for the wide breadth of this complication rate include a non-standard operative technique with respect to the skin incision and method of preparation in the subcutaneous tissues, as well as the use of different inclusion criteria or examination methods. Patient questionnaire studies tend to show a higher rate of meralgia paraesthetica, however, which could be explained by confounding of meralgia paraesthetica symptomatology with peri-incisional sensory changes [10]. Exclusion criteria in the studies tend to include complications not related to meralgia paraesthetica such as wound healing issues, which could, on the contrary, rather lead to under-reporting. Differing methods of examination with variable specificity and sensitivity, study designs, or inclusion criteria all contribute to the variability of the complication rates reported in the literature. On the other hand, most studies reporting on meralgia paresthetica have shown little to zero effect of this complication on the overall functional outcome of total hip replacement via the minimally invasive direct anterior approach in the long term [11].

Even with standard operative technique, the complication may not occur in all patients. In recent years, several anatomical studies have analysed the course and branching of the lateral femoral cutaneous nerve in representative cadaveric specimens [16,19,20,46]. The variability of coursing of the nerve occurs mostly at two sites—at the pelvic exit crossing the inguinal ligament, and in the area just distal to it, the proximal anterolateral thigh, where the variable branching pattern occurs.

In the 1997 cadaveric study with 104 hemipelves by Aszman et al. studying possible compression sites of the lateral femoral cutaneous nerve, five types of lateral femoral cutaneous nerve variations in the level of pelvis exit of the nerve were found, with respect to the ASIS and origin of the sartorius muscle, types A to E [19]. In type A, the nerve passes superficial and posterior to the anterior superior iliac spine and through the abdominal wall muscles. Type B describes the nerve coursing just medially to the anterior superior iliac spine and superficial to the sartorius muscle origin—this was found to be the most prevalent variant (27 percent). In type C (23 percent of specimens), the nerve passes medial to the anterior superior iliac spine deep to the inguinal ligament in a tendinous sheath, formed by an aponeurotic expansion of a variant medial tendinous origin of the sartorius muscle, depressing the inguinal ligament at contraction. Overall, 26 percent of specimens were identified as type D, where the nerve passes in-between the tendinous origin of the sartorius muscle and the iliopsoas muscle, where a thick septum also divides the muscles and connects to the deep fascia. Type E describes the most medial position of the nerve located in soft areolar tissue over the iliopsoas muscle; here, the inguinal ligament is a considerable distance from the nerve. Except for type A, the distribution was quite uniform among groups. Types A, B, and C were most likely to suffer from mechanical compression due to its superficiality or relationship to the inguinal ligament. With respect to the direct anterior approach total hip replacement, type A (incidence of 4%), passing lateral and posterior to the ASIS, and type C (incidence of 23%), encased in the common origin of sartorius and tensor fasciae latae muscles, would be most prone to injury during surgery.

In another, more recent, cadaveric study by Rudin et al., the at-risk location of pelvic exit of the nervus cutaneous femoris lateralis was found in 38% specimens where the nerve passed just superior or lateral to the spina iliaca anterior superior [16]. This study also showed the anatomical variability of lateral femoral cutaneous nerve in the plane of dissection during total hip replacement via the direct anterior approach. In all cases, the nerve ran within the deep layer of the subcutaneous fat tissue, under a weak fascia—an anatomical analogue of the abdominal Scarpa’s or Colles’ fascia. They grouped the branching pattern into three types—sartorius-type, posterior-type, and fan-type. Overall, 36% of specimens showed a dominant anterior branch of lateral femoral cutaneous nerve coursing along the lateral border of the sartorius muscle, with no branches crossing the proposed incision in the fascia over tensor fasciae latae muscle. Nine specimens (32%) showed a strong concomitant posterior branch equal or thicker than the anterior branch, consistently branching and coursing laterally and then distally immediately distal to the ASIS. It also ran with one or two fine vessels which could be used as landmarks for this branch. Overall, 32% showed a “fan-type” branching pattern, where multiple nerve branches of similar girth spread all over the anterolateral region of the thigh crossing over the tensor fasciae latae muscle and lateral border of the sartorius muscle at multiple points. Extrapolating from this, injury to the lateral femoral cutaneous nerve cannot be avoided in approximately one third of patients with the fan-type of lateral femoral cutaneous nerve. In the other two-thirds, a more lateral incision of the skin and fascial layers and careful dissection of subcutaneous tissues could be theoretically avoided. With proximal extension of the approach, the risk increases.

Our study shows the rate of lateral femoral cutaneous nerve injury is not a rare complication, with an incidence of 4.1%. All diagnoses were made by a single consulting neurology specialist. Most patients presented with a sensation of discomfort and fear. All of them were educated on the diagnosis and the likely long-term outcomes until satisfied. Despite the non-negligible rate of the early incidence of the complication, most cases resolved by 1 or 2 years after surgery without the need for other intervention.

**Figure 3:**
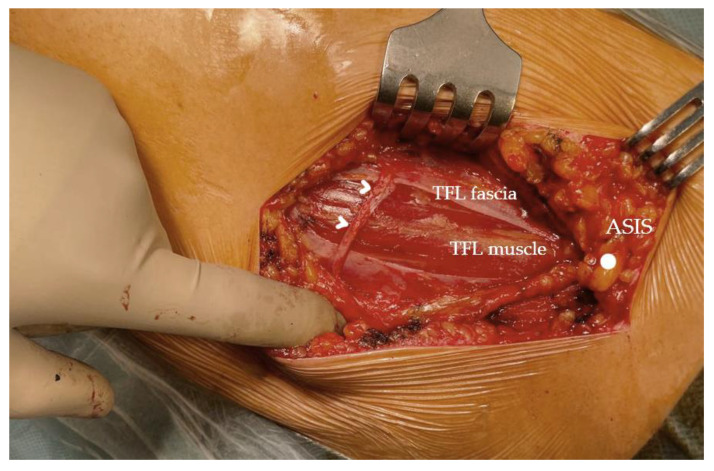
Perioperative photo of the direct anterior approach to a left hip dissected to the level of TFL. Shown here a case of a strong posterior branch of the LFCN, branching laterally and crossing the TFL muscle and the fascial incision at a rather distal position. Arrowheads—lateral femoral cutaneous nerve branch, TFL—tensor fasciae latae, ASIS—anterior superior iliac spine.

**Figure 4:**
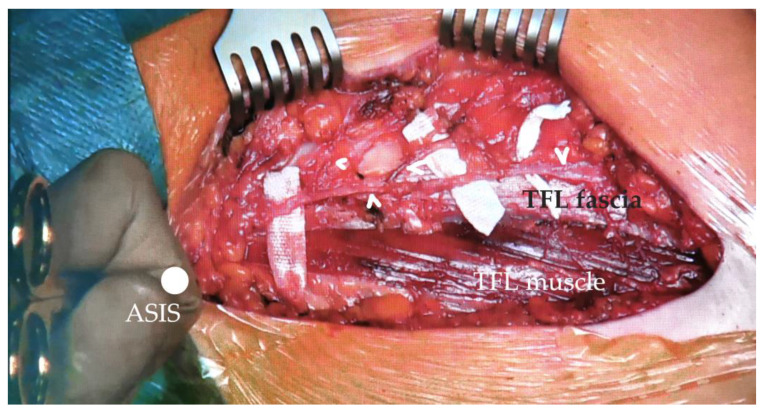
Perioperative photo of the direct anterior approach to a right hip dissected to the level of TFL. Shown here is a case of a fan-type branching of the LFCN with the anterior branches left intact, likely several small posterior branches were severed during fasciotomy of the TFL epimysium. Arrowheads—lateral femoral cutaneous nerve branches, TFL—tensor fasciae latae, ASIS—anterior superior iliac spine.

**Figure 5:**
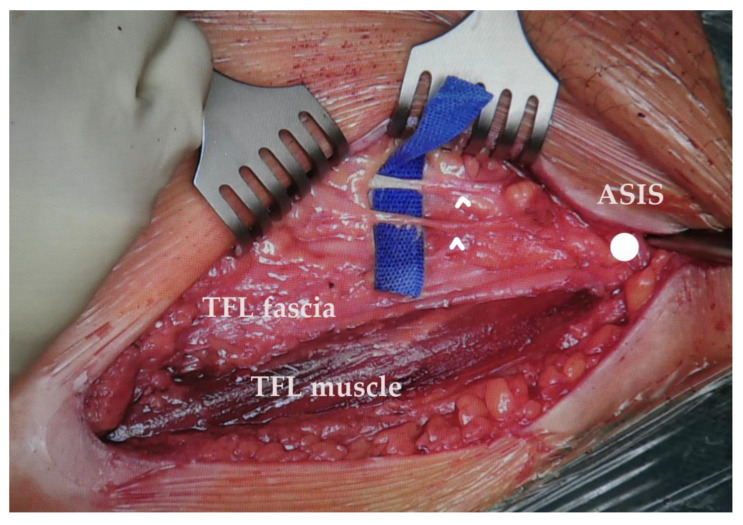
Perioperative photo of the direct anterior approach to a left hip dissected to the level of TFL. Shown here is a case of a sartorius type branching pattern of the LFCN with two similarly sized anterior branches of the LFCN, medial to the ASIS. No branches were found crossing the fascial incision. Arrowheads—lateral femoral cutaneous nerve branches, TFL—tensor fasciae latae, ASIS—anterior superior iliac spine.

According to the literature, the risk of meralgia paraesthetica is higher in patients with a history of other compressive neuropathies such as a previous carpal tunnel syndrome, higher body mass index (BMI), neurologic sequela of diabetes mellitus, and pregnancy [26,47]. The increased risk in the diabetic patient is hypothesized to be due to increased swelling of the nerve due to insufficient axoplasmic transport or due to a protein deficiency resulting in NaK-ATPase dysregulation, all resulting in increased susceptibility to nerve compression. Clothing could also play a factor [29]. Examples of branching patterns of the lateral femoral cutaneous nerve and its location with respect to the surgical plane depicted in Figure 3, Figure 4 and Figure 5.

Limitations of our study are using subjective signs of lateral femoral cutaneous nerve lesion, and a clinical diagnosis as opposed to radiological (e.g., ultrasound) or electrophysiological, as this was normal clinical practice during the study period. This can lead to underreporting of the complication rate of meralgia paresthetica. This is explained by the fact that lateral femoral cutaneous nerve injury has no effect on the functional outcome of total hip replacement, and only the subjectively significant lesions causing the patient discomfort or decrease of function would warrant investing further resources in the clinical practice. On the other hand, peri-incisional pain and discomfort could confound the results in the way of overestimating the complication rate. In this study, it was assumed the confounding risk of this kind was limited by examining the patient by both an orthopod and neurologist.

Furthermore, our study was retrospective, and no notes were found on the matter of identification the branching pattern types for all patients and correlating the three main types (according to Rudin) to the likelihood of developing meralgia paraesthetica. The possibility of incompleteness of medical records is also an option. By the same measure, comparing to the existing literature is difficult as study protocols, outcome measures and designs vary—prospective versus retrospective, or subjective patient outcome questionnaires versus objective testing. This results also in vastly different numbers in terms of meralgia paraesthetica rates after surgery [10,11,31,32].

Also, we observed only a single operative technique using the same skin incision and tissue dissection. Goulding showed a higher rate of MP in patients undergoing hip resurfacing as opposed to a total replacement [10]. Cadaveric dissection studies have shown a lower theoretical risk of LFCN injury via an anterolateral approach because of the lateral skin incision but also lowering the risk of LFCN injury in anterior approach with proximal shortening of the incision [48]. In one clinical study mentioning LFCN injury incidence in comparing direct anterior and a mini-anterolateral approach, they found no cases of MP [49]. The usual neurological complication mentioned in studies of anterolateral and lateral approaches are damage to the nervus gluteus superior, with an estimated average incidence of up to 77%, or more rarely, the femoral nerve [50,51]. In posterior approaches, the common neurological complication is sciatic nerve injury, with incidence ranging from 0.05% to 1.9% [52,53]. These, however rare, tend to have much more significantly negative functional outcome when comparing to the injury of the LFCN.

In future studies, we should adapt findings from the cadaveric studies and experimentally compare our approach with approaches utilizing the more lateral skin incision or lateral fasciotomy of the epimysium of the tensor fasciae latae or using a more distal window when opening the tensor fasciae latae fascia [54,55]. Also, using a prospective study protocol with preoperative ultrasound of the LCFN branching pattern as part of the preoperative planning could yield more trustworthy results.

## 5. Conclusions

This study showed a low, albeit significant, rate of meralgia paresthetica in our single-centre, single-surgeon retrospective study of a selected 2-year period. In the future, using a preoperative ultrasound to detect a specific branching pattern and adapting the surgical approach thusly could help us lower the rate of lateral femoral cutaneous nerve injury [18,54,55,56]. All patients undergoing total hip replacement via the direct anterior approach should receive thorough preoperative education about the possibility of iatrogenic meralgia paraesthetica. The surgeon should expect this complication as well as focus more care on the superficial dissection of direct anterior approach total hip replacement, more when considering extension of the approach, however not at the expense of risking proper bony preparation and implant placement.

## Figures and Tables

**Table 1:** Distribution of the symptomatic MP patients in the studied cohort and their progression over the follow-up period. FU = follow-up. Percentage of symptomatic patients out of all studied patients. Respective distribution of the symptom’s character—percentage point out of all the symptomatic patients.

FU	6 Weeks	6 Months	12 Months	24 Months
Symptomatic	17 (4.1%)	15 (3.5%)	7 (1.7%)	0
- pain	1	1	0	0
- numbness	12	10	4	0
- burning	1	1	1	0
- paraesthesia	5	3	2	0
- discomfort	6	2	0	0

## Data Availability

Dataset available upon request.

## References

[1] Moreau P. (2018). Minimally Invasive Total Hip Arthroplasty Using Hueter’s Direct Anterior Approach. Eur. J. Orthop. Surg. Traumatol..

[2] Rachbauer F., Kain M.S.H., Leunig M. (2009). The History of the Anterior Approach to the Hip. Orthop. Clin. N. Am..

[3] Wang Z., Bao H.W., Hou J.Z. (2019). Direct Anterior versus Lateral Approaches for Clinical Outcomes after Total Hip Arthroplasty: A Meta-Analysis. J. Orthop. Surg. Res..

[4] Rivera F., Comba L.C., Bardelli A. (2022). Direct Anterior Approach Hip Arthroplasty: How to Reduce Complications—A 10-Years Single Center Experience and Literature Review. World J. Orthop..

[5] Nairn L., Gyemi L., Gouveia K., Ekhtiari S., Khanna V. (2021). The Learning Curve for the Direct Anterior Total Hip Arthroplasty: A Systematic Review. Int. Orthop..

[6] Shen K., Feng E., Lin F., Weng Y., Chen J. (2022). Learning Curve of Total Hip Arthroplasty in Direct Anterior Approach without Requiring Corrective Osteotomy for Hip Dysplasia. Orthop. Surg..

[7] Peters R.M., Ten Have B.L.E.F., Rykov K., Van Steenbergen L., Putter H., Rutgers M., Vos S., Van Steijnen B., Poolman R.W., Vehmeijer S.B.W. (2022). The Learning Curve of the Direct Anterior Approach Is 100 Cases: An Analysis Based on 15,875 Total Hip Arthroplasties in the Dutch Arthroplasty Register. Acta Orthop..

[8] Hasija R., Kelly J.J., Shah N.V., Newman J.M., Chan J.J., Robinson J., Maheshwari A.V. (2018). Nerve Injuries Associated with Total Hip Arthroplasty. J. Clin. Orthop. Trauma.

[9] Vajapey S.P., Morris J., Lynch D., Spitzer A., Li M., Glassman A.H. (2020). Nerve Injuries with the Direct Anterior Approach to Total Hip Arthroplasty. JBJS Rev..

[10] Goulding K., Beaulé P.E., Kim P.R., Fazekas A. (2010). Incidence of Lateral Femoral Cutaneous Nerve Neuropraxia after Anterior Approach Hip Arthroplasty. Clin. Orthop. Relat. Res..

[11] Bhargava T., Goytia R.N., Jones L.C., Hungerford M.W. (2010). Lateral Femoral Cutaneous Nerve Impairment after Direct Anterior Approach for Total Hip Arthroplasty. Orthopedics.

[12] Restrepo C., Parvizi J., Pour A.E., Hozack W.J. (2010). Prospective Randomized Study of Two Surgical Approaches for Total Hip Arthroplasty. J. Arthroplast..

[13] Homma Y., Baba T., Sano K., Ochi H., Matsumoto M., Kobayashi H., Yuasa T., Maruyama Y., Kaneko K. (2016). Lateral Femoral Cutaneous Nerve Injury with the Direct Anterior Approach for Total Hip Arthroplasty. Int. Orthop..

[14] Grob K., Manestar M., Ackland T., Filgueira L., Kuster M.S. (2014). Potential Risk to the Superior Gluteal Nerve during the Anterior Approach to the Hip Joint an Anatomical Study. J. Bone Jt. Surg.—Am. Vol..

[15] Starke V., Stofferin H., Mannschatz S., Hörmann R., Dammerer D., Thaler M. (2021). The Anatomical Course of the Superior Gluteal Nerve with Regard to the Direct Anterior Approach for Primary and Revision Total Hip Arthroplasty. J. Arthroplast..

[16] Rudin D., Manestar M., Ullrich O., Erhardt J., Grob K. (2016). The Anatomical Course of the Lateral Femoral Cutaneous Nerve with Special Attention to the Anterior Approach to the Hip Joint. J. Bone Jt. Surg.—Am. Vol..

[17] Chang K.V., Mezian K., Naňka O., Wu W.T., Lou Y.M., Wang J.C., Martinoli C., Özçakar L. (2018). Ultrasound Imaging for the Cutaneous Nerves of the Extremities and Relevant Entrapment Syndromes: From Anatomy to Clinical Implications. J. Clin. Med..

[18] Majkrzak A., Johnston J., Kacey D., Zeller J. (2010). Variability of the Lateral Femoral Cutaneous Nerve: An Anatomic Basis for Planning Safe Surgical Approaches. Clin. Anat..

[19] Aszmann O.C., Dellon E.S., Dellon A.L. (1997). Anatomical Course of the Lateral Femoral Cutaneous Nerve and Its Susceptibility to Compression and Injury. Plast. Reconstr. Surg..

[20] Carai A., Fenu G., Sechi E., Crotti F.M., Montella A. (2009). Anatomical Variability of the Lateral Femoral Cutaneous Nerve: Findings from a Surgical Series. Clin. Anat..

[21] Ropars M., Morandi X., Huten D., Thomazeau H., Berton E., Darnault P. (2009). Anatomical Study of the Lateral Femoral Cutaneous Nerve with Special Reference to Minimally Invasive Anterior Approach for Total Hip Replacement. Surg. Radiol. Anat..

[22] Moritz T., Prosch H., Berzaczy D., Happak W., Lieba-Samal D., Bernathova M., Auff E., Bodner G. (2013). Common Anatomical Variation in Patients with Idiopathic Meralgia Paresthetica: A High Resolution Ultrasound Case-Control Study. Pain. Physician.

[23] Tomaszewski K.A., Popieluszko P., Henry B.M., Roy J., Sanna B., Kijek M.R., Walocha J.A. (2016). The Surgical Anatomy of the Lateral Femoral Cutaneous Nerve in the Inguinal Region: A Meta-Analysis. Hernia.

[24] Standring S. (2015). Gray’s Anatomy: The Anatomical Basis of Clinical Practice.

[25] Gray H., Lewis W., Gray H. (1918). Anatomy of the Human Body.

[26] Ivins G.K. (2000). Meralgia Paresthetica, the Elusive Diagnosis. Ann. Surg..

[27] Roth V.K. (1896). Meralgia Paræesthetica. Von Dr. Wladimir K. Roth. Williams and Norgate. London: 1895, pp. 24. J. Ment. Sci..

[28] de Ruiter G.C.W., Oosterhuis J.W.A., Vissers T.F.H., Kloet A. (2023). Unusual Causes for Meralgia Paresthetica: Systematic Review of the Literature and Single Center Experience. Neurosurg. Rev..

[29] Moucharafieh R., Wehbe J., Maalouf G. (2008). Meralgia Paresthetica: A Result of Tight New Trendy Low Cut Trousers (‘taille Basse’). Int. J. Surg..

[30] Lee S.H., Shin K.J., Gil Y.C., Ha T.J., Koh K.S., Song W.C. (2017). Anatomy of the Lateral Femoral Cutaneous Nerve Relevant to Clinical Findings in Meralgia Paresthetica. Muscle Nerve.

[31] Peters G., Larner A.J. (2006). Meralgia Paresthetica Following Gynecologic and Obstetric Surgery. Int. J. Gynecol. Obstet..

[32] Kavanagh D., Connolly S., Fleming F., Hill A.D.K., McDermott E.W., O’Higgins N.J. (2005). Meralgia Paraesthetica Following Open Appendicectomy. Ir. Med. J..

[33] Parisi T.J., Mandrekar J., Dyck P.J.B., Klein C.J. (2011). Meralgia Paresthetica: Relation to Obesity, Advanced Age, and Diabetes Mellitus. Neurology.

[34] Williams P.H., Trzil K.P. (1991). Management of Meralgia Paresthetica. J. Neurosurg..

[35] Khalil N., Nicotra A., Rakowicz W. (2012). Treatment for Meralgia Paraesthetica. Cochrane Database Syst. Rev..

[36] Hurdle M.F., Weingarten T.N., Crisostomo R.A., Psimos C., Smith J. (2007). Ultrasound-Guided Blockade of the Lateral Femoral Cutaneous Nerve: Technical Description and Review of 10 Cases. Arch. Phys. Med. Rehabil..

[37] De Ruiter G.C.W., Wurzer J.A.L., Kloet A. (2012). Decision Making in the Surgical Treatment of Meralgia Paresthetica: Neurolysis versus Neurectomy. Acta Neurochir..

[38] Benezis I., Boutaud B., Leclerc J., Fabre T., Durandeau A. (2007). Lateral Femoral Cutaneous Neuropathy and Its Surgical Treatment: A Report of 167 Cases. Muscle Nerve.

[39] Payne R., Seaman S., Sieg E., Langan S., Harbaugh K., Rizk E. (2017). Evaluating the Evidence: Is Neurolysis or Neurectomy a Better Treatment for Meralgia Paresthetica?. Acta Neurochir..

[40] MacKay M.D., Mudreac A., Varacallo M. Anatomy, Abdomen and Pelvis, Camper Fascia. https://www.ncbi.nlm.nih.gov/books/NBK482246/.

[41] Matta J.M., Sah A.P. (2022). Anterior Hip Replacement: From Origin to Current Advanced Techniques.

[42] Nogler M. (2022). The History of the Direct Anterior Approach in Innsburck. Anterior Hip Replacement: From Origin to Current Advanced Techniques.

[43] Corten K., Holzapfel B.M. (2021). Direct Anterior Approach for Total Hip Arthroplasty Using the “Bikini Incision”. Oper. Orthop. Traumatol..

[44] Leunig M., Hutmacher J.E., Rüdiger H.A., Naal F.D., Ricciardi B.F., Impellizzeri F.M. (2018). Skin Crease “bikini” Incision for the Direct Anterior Approach in Total Hip Arthroplasty. Bone Jt. J..

[45] Sang W., Xue S., Xu Y., Liu Y., Zhu L., Ma J. (2021). Bikini Incision Increases the Incidence of Lateral Femoral Cutaneous Nerve Injury in Direct Anterior Approach Hip Arthroplasty: A Prospective Ultrasonic, Electrophysiological, and Clinical Study. J. Arthroplast..

[46] Schopper C., Traxler H., Schauer B., Hipmair G., Gotterbarm T., Luger M. (2021). Minimally Invasive Approaching in Hip Surgery—An Anatomical Investigation of 20 Specimens. Medicina.

[47] Van Slobbe A.M., Bohnen A.M., Bernsen R.M.D., Koes B.W., Bierma-Zeinstra S.M.A. (2004). Incidence Rates and Determinants in Meralgia Paresthetica in General Practice. J. Neurol..

[48] Ukai T., Suyama K., Hayashi S., Omura H., Watanabe M. (2022). The Anatomical Features of the Lateral Femoral Cutaneous Nerve with Total Hip Arthroplasty: A Comparative Study of Direct Anterior and Anterolateral Supine Approaches. BMC Musculoskelet. Disord..

[49] Herndon C.L., Drummond N., Sarpong N.O., Cooper H.J., Shah R.P., Geller J.A. (2020). Direct Anterior versus Mini-Anterolateral Approach for Primary Total Hip Arthroplasty: Early Postoperative Outcomes and Complications. Arthroplast. Today.

[50] Beaulieu M.A., Laurin C.A. (1990). Gluteal Nerve Damage Following Total Hip Arthroplasty: A Prospective Analysis. J. Arthroplast..

[51] Chomiak J., Huráček J., Dvořák J., Dungl P., Kubeš R., Schwarz O., Munzinger U. (2015). Lesion of Gluteal Nerves and Muscles in Total Hip Arthroplasty through 3 Surgical Approaches. An Electromyographically Controlled Study. HIP Int..

[52] Yang I.-H. (2014). Neurovascular Injury in Hip Arthroplasty. Hip Pelvis.

[53] Brown G.D., Swanson E.A., Nercessian O.A. (2008). Neurologic Injuries after Total Hip Arthroplasty. Am. J. Orthop..

[54] Tanabe H., Baba T., Ozaki Y., Yanagisawa N., Banno S., Watari T., Homma Y., Nagao M., Kaneko K., Ishijima M. (2022). Lateral versus Conventional Fasciotomy for Prevention of Lateral Femoral Cutaneous Nerve Injury in Total Hip Arthroplasty with Direct Anterior Approach: A Study Protocol for a Dual-Center, Double-Blind, Randomized Controlled Trial. Trials.

[55] Ozaki Y., Baba T., Homma Y., Tanabe H., Ochi H., Bannno S., Watari T., Kaneko K. (2018). Preoperative Ultrasound to Identify Distribution of the Lateral Femoral Cutaneous Nerve in Total Hip Arthroplasty Using the Direct Anterior Approach. SICOT J..

[56] Ozaki Y., Homma Y., Sano K., Baba T., Ochi H., Desroches A., Matsumoto M., Yuasa T., Kaneko K. (2016). Small Femoral Offset Is a Risk Factor for Lateral Femoral Cutaneous Nerve Injury during Total Hip Arthroplasty Using a Direct Anterior Approach. Orthop. Traumatol. Surg. Res..

